# Management of Hyperuricemia in Patients with Chronic Kidney Disease: a Focus on Renal Protection

**DOI:** 10.1007/s11906-020-01116-3

**Published:** 2020-10-31

**Authors:** Jan T. Kielstein, Roberto Pontremoli, Michel Burnier

**Affiliations:** 1 Medical Clinic V, Nephrology, Rheumatology, Blood Purification, Academic Teaching Hospital Brauchweig, Brunswick, Germany; 2 Università degli Studi and I.R.C.C.S. Ospedale Policlinico San Martino, Genoa, Italy; 3 Service of Nephrology and Hypertension Department of Medicine Lausanne University Hospital, Centre Hospitalier Universitaire Vaudois, University of Lausanne Switzerland, Lausanne, Switzerland

**Keywords:** Hyperuricemia, Hypertension, Chronic kidney disease, Urate-lowering therapy, Renal protection, Allopurinol, Febuxostat

## Abstract

**Purpose of Review:**

In chronic kidney disease (CKD), plasma uric acid levels are increased because of the decrease in glomerular filtration rate. However, in addition to CKD, hyperuricemia is frequently associated with a number of other conditions such as hypertension, type 2 diabetes, obesity, and heart failure, overweight, and cardiovascular disease.

**Recent Findings:**

It is now becoming increasingly clear that, in many clinical conditions, elevated levels of uric acid have a much greater role beyond just causing gout. The present review will summarize current knowledge on the relation between hyperuricemia, CKD, and existing comorbidities, as well as the mechanisms of uric acid–related renal damage. In addition, the role and evidence for urate-lowering therapy in prevention and cardiovascular protection in CKD patients is discussed with a focus on allopurinol and febuxostat. To date, several clinical studies have provided evidence that urate-lowering therapy may help to prevent and delay the decline of renal function in patients with CKD.

**Summary:**

Use of a xanthine oxidase inhibitor should be considered in patients who are at high renal risk and/or with declining renal function in the presence of hyperuricemia with and without deposition, although additional studies are warranted to define treatment targets. Notwithstanding, the possibility to delay deterioration of renal function in patients with CKD merits consideration.

## Introduction

**Fig. 1:**
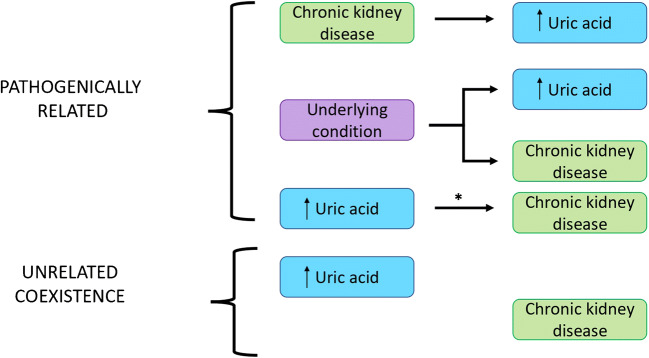
Possible relations between serum uric acid and chronic kidney disease (from [4] with permission). The asterisk indicates a high level of evidence

Uric acid is the end product of purine metabolism in humans. Two-thirds of uric acid circulating in the blood is excreted by the kidneys and one-third by the gastrointestinal tract. In the general population, results from the Framingham Heart Study have shown that, with age, serum uric levels remain relatively stable in men, whereas in women, serum uric acid levels gradually increase from the fourth to the seventh decades of life [1]. Thus, there is an age-dependent, hormone-dependent increase in uric acid levels in women, but not in men. In chronic kidney disease (CKD), plasma uric acid levels are increased because of the decrease in glomerular filtration rate. The main clinical consequence of hyperuricemia is gout with or without deposition. However, in addition to CKD, hyperuricemia is frequently associated with a number of other conditions including hypertension, type 2 diabetes, obesity, heart failure, and cardiovascular diseases [2]. In these clinical situations, hyperuricemia was considered initially as a passive bystander or a simple consequence of these comorbidities, but today, evidence suggests that hyperuricemia might have other clinical implications than just inducing gout in these disorders. In CKD, it is not easy to evaluate the causal influence of uric acid on the disease progression because it is difficult to ascertain if hyperuricemia preceded CKD or whether the reverse is true [3]. The possible relations between serum uric acid and chronic kidney disease are illustrated in Fig. 1 [4•].

The prevalence of hyperuricemia can be considered high worldwide. Using data from the NHANES survey 2007–2008, Zhu et al. reported a prevalence of hyperuricemia of 21% in the US general population, and this percentage appears to be increasing over the past two decades [5]. Hyperuricemia with deposition was also found to markedly increase with age in both males and females in a study from Italy [6]. Over the age of 75 years, around 40% of males had hyperuricemia, and roughly one in 20 had gout. Of interest, the recent increase in the prevalence of hyperuricemia has been related with the global increase in the prevalence of overweight and obesity and the increased consumption of foods rich in purines and alcohol and soft drinks sweetened with fructose [7].

### The Link Between Hyperuricemia and CKD

#### General Population

There is no doubt that hyperuricemia and gout increase in parallel with declining renal function. In the German Chronic Kidney Disease (GCKD) study, the overall prevalence of gout was 24.3% and increased from 16.0% in those with eGFR ≥ 60 ml/min/1.73 m^2^ to 35.6% in those with eGFR < 30 ml/min/1.73 m^2^ [8]. Numerous experimental and clinical studies have investigated the association between hyperuricemia and the development of future CKD [1, 9]. The relationship between uric acid and subsequently reduced kidney function has been examined in 13,338 participants with intact kidney function in two community-based cohorts, the Atherosclerosis Risks in Communities and the Cardiovascular Health Study in the USA [10]. In this analysis, an elevated serum uric acid level was an independent, albeit modest, risk factor for incident kidney disease in a generalizable US population over a lengthy follow-up period. Juraschek et al., using data from the NHANES survey, showed that hyperuricemia is significantly more frequent in individuals with hypertension and reduced glomerular filtration rates [11]. The prevalence of hyperuricemia also increased with decreasing renal function: 11–13% among individuals with an eGFR ≥ 90 ml/min/1.73 m^2^ versus 64–78% among individuals with an eGFR between 15 and 29 ml/min/1.73 m^2^ [11]. Hsu et al., in a study on > 175,000 individuals with a 25-year follow-up, found that elevated serum uric acid was an independent risk factor for end-stage renal disease (HR 2.14) [12]. Similar results were reported by Chonchol et al., where uric acid levels were associated with CKD and its progression [13]. The possible relation between hyperuricemia and incident CKD was further confirmed in a large meta-analysis on more than 190,000 individuals [14]. Hyperuricemia was an independent predictor for diagnosis of new CKD (OR 2.35), which increased with a longer follow-up. A longitudinal study further demonstrated that the proportion of individuals with increasingly higher levels of hyperuricemia increased steadily as eGFR decreased; moreover, the risk of progression to end-stage renal disease (ESRD) increased by 7% for each 1 mg/dl increase in baseline uric acid levels [15]. Using a novel method of data analysis, a semiparametric group-based trajectory model, the same authors reported that high serum uric acid levels are indeed associated with accelerated kidney failure and all-cause mortality in a cohort of 5090 CKD patients [16]. A population-based cohort study in 3605 participants followed for 5.2 years found that participants with persistently high hyperuricemia had a significantly higher risk of developing CKD than those with persistently low serum uric acid (HR 2.05) [17]. Taken together, these observations suggest that, while it is recognized that there has been some debate about the role of uric acid in CKD, data from a wide range of experimental, clinical, and epidemiologic studies tend to support the hypothesis of a pathogenic role for uric acid in CKD [10, 18–20].

#### Hyperuricemia, CKD, and Existing Comorbidities

The relation between uric acid and kidney failure has also been examined in individuals with existing CKD. As one example, Srivastava studied 3885 individuals with CKD stages 2–4 with a median follow-up of 7.9 years [21]. In that study, it was found that elevated uric acid was independently associated with kidney failure in patients with eGFR ≥ 45 ml/min/1.73 m^2^, but not in those with eGFRs < 30 ml/min/1.73 m^2^, and that there was also a J-shaped curve considering uric acid levels and all-cause mortality. Some studies did not find any association between hyperuricemia and CKD progression. This was the case in the Mild to Moderate Kidney Disease study, in which patients with non-diabetic CKD (*n* = 177) were followed for a 7-year period and hyperuricemia was not an independent predictor of CKD progression after adjustment for GFR and proteinuria [22]. In the Modification of Diet in Renal Disease study which followed 838 patients with CKD stages 3–4 for 10 years, investigators did not find any association between hyperuricemia and the development of end-stage kidney disease, although the risk of all-cause and cardiovascular mortality was significantly higher in patients with a uric acid > 500 μmol/l [23].

Several studies have also examined the relation between hyperuricemia and the development of CKD in patients with type 2 diabetes (T2D). A longitudinal study of 62,830 of patients with T2D using the database of the Italian Association of Clinical Diabetologists network found that, at a 4-year follow-up, 7.9% of patients developed GFR < 60 ml/min per 1.73 m^2^ with normoalbuminuria and 14.1% had albuminuria with eGFR ≥ 60 ml/min/1.73 m^2^, while 2.0% had albuminuria with eGFR < 60 ml/min/1.73 m^2^ [24]. Furthermore, the proportion of patients with eGFR < 60 ml/min/1.73 m^2^ increased with uric acid quintiles. Compared with the lowest quintile, relative risk ratios were 1.46, 1.44, 1.95, and 2.61 for the second, third, fourth, and fifth quintiles, respectively. It was thus concluded that mild hyperuricemia is strongly associated with the risk of CKD in patients with T2D.

In 2020, Kaewput et al. published the results of a study in almost 8500 patients with T2D and found that uric acid was among the risk factors associated with progression to CKD stage 5 with a hazard ratio of 1.24 per 1 mg/dl increase (95% CI 1.13–1.36) [25]. A meta-analysis on 25,453 patients with CKD concluded that patients with the highest serum uric acid levels had a significantly higher risk for mortality (HR, 1.52; 95% CI, 1.33–1.73) vs. those with the lowest levels. In addition, an increase of 1 mg/dl in serum uric acid was associated with an 8% increased risk of mortality (HR, 1.08; 95% CI, 1.04–1.11) [26].

The risk of developing hypertension is greater in subjects with hyperuricemia and the risk augments with age. Increased serum uric acid is significantly associated with the development of new-onset primary hypertension in children [34]. In a large meta-analysis of 25 studies including 97,824 participants, the elevated uric acid level predicted systemic hypertension [27].

Hyperuricemia has been further linked to an increased risk for stroke as well as other cardiometabolic risk factors including increased BMI and triglycerides in individuals with T2D [28]. Moreover, patients with T2D and hyperuricemia and decreased urinary uric acid excretion are at even more risk for developing CKD; in this particular subgroup, the prevalence of CKD is reported to be 47.8% [29]. Inhibitors of sodium/glucose co-transporter-2 (SGLT2) are now used to treat T2D as they lower blood glucose by increasing renal elimination of glucose. Of interest, SGLT2 inhibitors also lower serum uric acid, which may in part explain the finding that this class of drugs is also associated with improved cardiorenal risk, i.e., reduces the risk of cardiovascular events and slows progression of CKD in patients with T2D [30]. However, the intracellular accumulation of uric acid might be responsible for acute kidney injury in patients treated with SGLT-2 inhibitors [31]. In a large meta-analysis on 13,650 patients, SGLT2 inhibitors were found to decrease serum uric acid from − 37 to − 42 μmol/l, which may be beneficial for patients with T2D and hyperuricemia [32].

Hyperuricemia is common in renal transplant recipients [33]. Following transplantation, lower eGFR levels have been correlated with higher levels of serum uric acid [34]. Moreover, hyperuricemia is an adverse effect of calcineurin inhibitors, mainly ciclosporin. However, the impact of eGFR on graft survival and mortality remains a subject of debate. Studies have reported that hyperuricemia is not associated with loss of graft or increased rates of mortality [34]. In a retrospective cohort analysis in almost 3000 kidney transplant patients, Kim et al. found that normal to low levels of serum uric acid during the first-year post-transplantation and over a 5-year follow-up period were associated with better allograft outcomes [35]. However, this observation may reflect that patients with an excellent graft function at 5 years have a better outcome, with the uric acid level being a marker of renal function. Donor age may also have a substantial effect on allograft function, even if those receiving a graft from an older donor may still have adequate graft function [36]. Of interest, a study on 48 and 33 patients receiving febuxostat and allopurinol, respectively, reported that the drugs showed no clinically significant differences on allograft function and both were well tolerated, even if febuxostat was associated with more rapid urate lowering [37]. In patients with ESRD undergoing hemodialysis, a small study on 120 patients reported that hyperuricemia was a predictor of higher mortality [38].

### Mechanisms of Uric Acid–Related Renal Damage

**Fig. 2:**
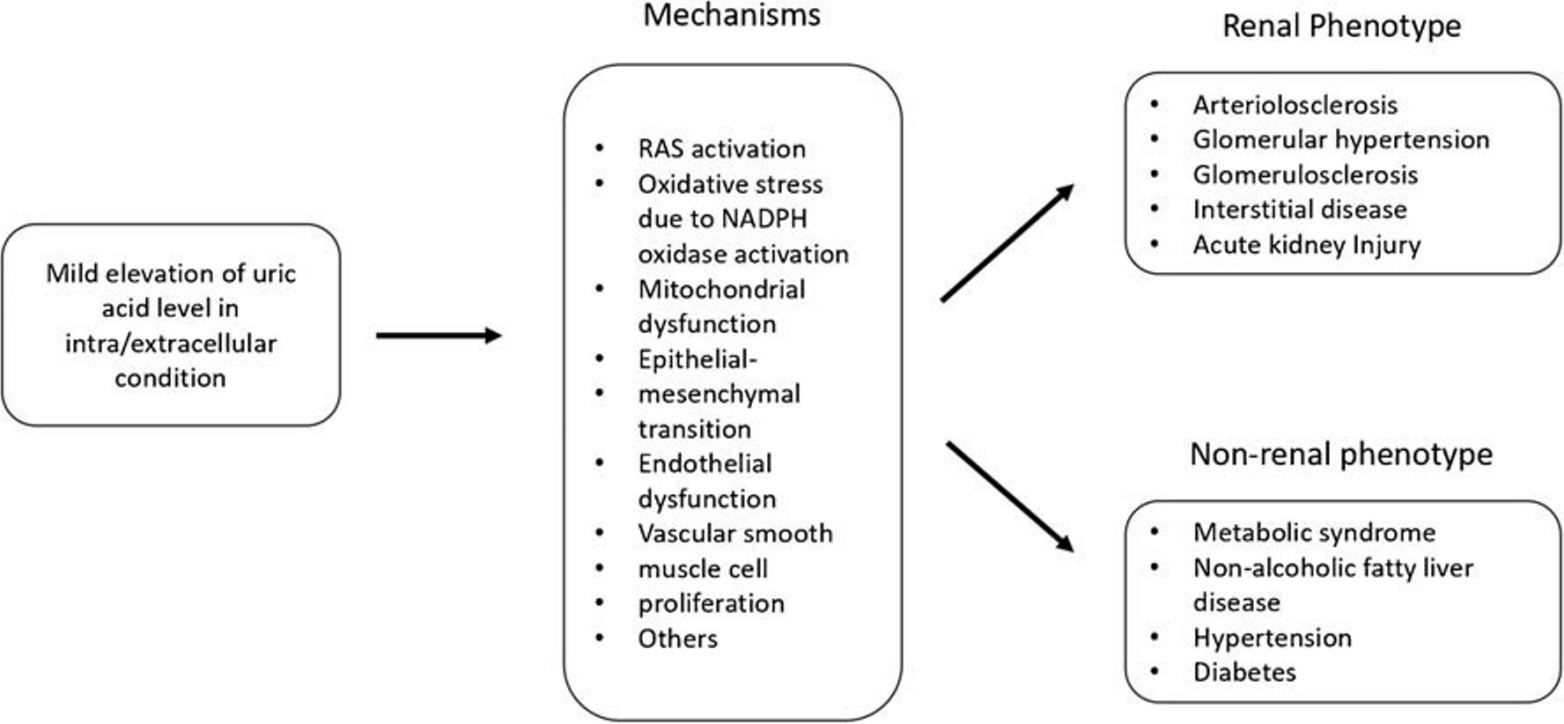
Mechanisms by which uric acid may contribute to the development of renal and non-renal diseases (from [39] with permission)

Multiple mechanisms have been related to renal damage caused by uric acid. The main mechanisms by which uric acid contributes to the development of renal and non-renal diseases have been summarized by Johnson et al. as shown in Fig. 2 [39].

Uric acid is known to act as a mediator of hypertension through its effects on endothelial function and impaired production of nitric oxide [40••]. Initially, hyperuricemia was thought to cause CKD by the deposition of urate crystals in the renal interstitium, the so-called urate nephropathy. However, it was concluded that gout and hyperuricemia per se did not cause renal insufficiency, but that the likeliest malefactor was the association with hypertension [41]. One hypothesis was that the deposition of uric acid crystals in the renal interstitium is favored by the development of hypertension-induced areas of ischemia. Thus, hypertension might be the initial trigger leading to subclinical renal damages thereby inducing hyperuricemia and the development of a vicious circle linking hypertension, hyperuricemia, and renal damages.

Today, another hypothesis is arising suggesting that hyperuricemia is a contributor to hypertension, which leads to renal injury [42]. Investigations in animal models have shown that hyperuricemia causes hypertension through activation of both vasoactive and inflammatory processes that have multiple effects that include sodium retention and vascular constriction, which in turn leads to hypertension [43]. This was shown by the observation that, in rats, administration of an uricase inhibitor leads to hyperuricemia and development of hypertension [44]. Indeed, rats made hyperuricemic displayed all of classic findings seen in essential hypertension, such as prominent vasoconstriction of the afferent arteriole and decreased renal blood flow, with relative preservation of GFR [45]. Histologic analyses documented lesions that were similar to those seen in hypertension, with the presence of arteriolosclerosis and tubule-interstitial injury [44]. While hyperuricemia has been reported to affect renal hemodynamics in rat models, intrarenal hemodynamic parameters have also been examined in humans, where serum uric acid levels significantly correlated with vascular resistance at the afferent, but also efferent, arteriole, suggesting that hyperuricemia may be associated to dysfunction of glomerular perfusion [46].

Uric acid has also been found to have a large variety of cellular effects in vitro. Soluble uric acid can enter cells via specific transporters where it stimulates signaling mechanisms that lead to the release of inflammatory mediators, vasoconstrictor molecules, growth factors, and oxidants as well as inducing mitochondrial dysfunction (reviewed in [43]). It has also been reported that uric acid induces oxidative stress in a variety of cell types [45, 47, 48], even if the role of uric acid in oxidative stress as a potent antioxidant remains controversial [7, 43]. One possibility is that uric acid induces oxidative stress through activation of nicotinamide adenine dinucleotide phosphate oxidase [47].

One of the main mechanisms through which hyperuricemia leads to hypertension and CKD may be via activation of the renin-angiotensin-aldosterone system (RAAS) [42]. In this scenario, elevated levels of uric acid stimulate expression of renin by myoepithelial cells in the afferent arteriole, and further uric acid also activates prorenin receptors in proximal tubular cells that stimulate the angiotensin system in the kidney. Systemic and renal vasoconstriction as well as reduced renal plasma flow would be the result of activation of RAAS and other vasoconstrictors, and suppression of vasodilators such as nitric oxide. This could lead to the development of afferent arteriolar hypertrophy and an inadequate afferent vasoconstrictor response thus transmitting systemic pressure to glomeruli, promoting progression of CKD. Urate may also stimulate NADPH oxidase to increase oxidative stress, leading to the aforementioned mitochondrial dysfunction, secretion of pro-inflammatory cytokines, and propagation of vascular smooth muscle cells. Lastly, it is possible that urate crystals can cause tubular damage through inflammation mediated by the inflammasome or by direct physical mechanisms.

Innate immune pathways are also increasingly recognized to be important in the pathogenesis of hyperuricemia with deposition, and in particular activation of the NLRP3 inflammasome, which leads to the release of IL-1β and other pro-inflammatory cytokines [49]. The orchestration of this pro-inflammatory cascade involves multiple intracellular and extracellular receptors and enzymes interacting with environmental influences that modulate the inflammatory state [49]. The urate-induced inflammasome pathway is comprised of urate crystal uptake into intracellular lysosomes and subsequent lysosomal rupture with production of mitochondrial reactive oxygen species (ROS), which activates the NLRP3 inflammasome [50]. In addition, increased cellular urate, directly or indirectly via xanthine oxidase-induced oxidative stress, may be associated with inflammasomes [50]. There are two mechanisms that underlie the role of inflammation in relation to uric acid metabolism [51]. The first is inflammasome activation by uric acid crystallization, and the second involves superoxide free radicals generated by xanthine oxidase (XO) [51]. The inflammasome concept was proposed to involve multiple proteins and to control the cleavage of prointerleukin 1 (IL-1) [51]. Excess metabolites, such as MSU crystals, are involved in activation of inflammasomes; inflammatory responses occurring via inflammasomes have been demonstrated to be linked to the onset and progression of hyperuricemia with deposition [51]. While the UA crystallization mechanism appears to be dependent on high levels of uric acid, the generation of superoxide free radicals produced by XO may not entirely reflect the levels of serum uric acid as XO activity leads to the production of ROS [51]. Taken together, there are multiple, well-studied mechanisms and pathways through which uric acid can affect renal function and lead to renal damage when elevated over time. Moreover, while out of the scope of the present review, as both adverse renal and cardiovascular effects are not independent of one another [52–55], uric acid is considered to adversely affect cardiovascular health through many of the same mechanisms discussed above.

### Role of Urate-Lowering Therapy in Prevention of CKD

In the German CKD study, only ~ 70% of individuals with self-reported gout were taking gout medications [8]. Hence, based on the available evidence, gout patients with CKD should be treated. One problem is that the dose of urate-lowering therapy (ULT), when using allopurinol, has to be tailored to the renal function. In the German cohort, patients with lower doses of allopurinol had higher serum urate levels than patients with higher (than recommended) allopurinol doses [56•]. This problem can be overcome if febuxostat is used, as it is excreted in a dual way via the liver and the kidney. Thus, the question arises if hyperuricemia per se should be treated in CKD patients. Indeed, a wealth of data on the link between hyperuricemia and CKD begs the question of whether ULT is a valid preventive-therapeutic strategy to prevent CKD and/or its progression. In individuals with normal renal function, and especially in high-risk patients at such as those with T2D, hypertension, and cardiovascular risk factors, primary prevention with ULT has the potential to prevent kidney damage and a decline in renal function. In patients with preexisting CKD, ULT might help in delaying progression to ESRD.

A large number of studies have been carried out to investigate the effects of ULT on CKD progression (reviewed in [42, 57, 58]). While many trials have reported that ULT has a benefit on renal outcomes, others found that ULT provided no benefit [59]. There are several explanations for the observed variability of ULT on CKD in clinical trials [42]. One possibility is that ULT does not provide a consistent benefit in all CKD patients. Another point is that available trials are highly heterogeneous in terms of the population being studied with different mean ages and stages of CKD of patients included. In addition, studies differ by their profile of comorbidities such as hypertension and T2D as well as by in the dose of ULT received and duration of treatment; many are also not randomized clinical trials. All of these factors have the potential to affect not only renal function at baseline but also its progression. The vast majority of trials have investigated allopurinol or febuxostat as ULT.

#### Allopurinol

Twelve randomized clinical studies have examined renal outcomes with allopurinol as ULT in hyperuricemic patients with CKD [60]. The trial by Goicoechea et al. was likely the first to examine the effects of allopurinol on progression of CKD and cardiovascular risk [61••]. In that prospective, randomized trial of 113 patients with eGFR < 60 ml/min, patients were randomized to allopurinol (*n* = 57) or to continue their usual therapy (*n* = 56); after 24 months, eGFR was decreased by 3.3 ± 1.2 ml/min/1.73 m^2^ in the control group and by 1.3 ± 1.3 ml/min/1.73 m^2^ in those treated with allopurinol (*p* = 0.018). However, the sample size was small, and this single-center study was not double-blind.

In 2014, Bose et al. carried out a meta-analysis of 8 trials involving 476 participants, noting that there was substantial heterogeneity in baseline kidney function, underlying causes of CKD, and length of follow-up across studies [62]. It was reported that there was no change in eGFR with active treatment vs. controls in 5 studies (*n* = 346), while it did abrogate increases in serum creatinine in 3 trials (*n* = 130). In another meta-analysis of 19 randomized trials in 992 patients with stage 3–5 CKD, allopurinol was associated with a modestly better eGFR compared with controls (mean difference 3.2 ml/min/1.73 m^2^) [63].

#### Febuxostat

At least 11 studies have investigated the effects of febuxostat on renal function in patients with CKD [64•]. Among them, seven lasted 6 months or more; in terms of patient inclusion criteria, 4 trials mention hyperuricemia and 2 asymptomatic hyperuricemia, and 2 trials enrolled patients with hyperuricemia and excluded those with gout, and 1 trial each for gout, gout or history of gout, and gout or asymptomatic hyperuricemia. A meta-analysis of those trials, which included 1137 patients, found that febuxostat was associated with a significant reduction in serum uric acid [64•]. Considering changes in renal function vs. placebo, febuxostat was associated with higher eGFR (weighted mean difference, 2.36 ml/min/1.73 m^2^; 95% CI, − 1.62 to 6.33), but the difference was not significant. However, among patients with stage 3 and 4 CKD, significantly higher eGFR was found in those treated with febuxostat vs. placebo (weighted mean difference, 3.66 ml/min/1.73 m^2^; 95% CI, 0.76 to 6.55). The renal benefits of febuxostat were also significant in studies lasting more than 6 months. No significant differences in major complication or death were seen between the febuxostat and control groups. Considering these findings, the meta-analysis concluded that, in addition to lowering serum urate, febuxostat had a reno-protective effect in patients with CKD. The occurrence of renal events in patients with hyperuricemia treated with febuxostat and those treated with conventional therapy with lifestyle modification was also investigated in the FREED trial, a multicenter, prospective, randomized open-label, blinded endpoint study conducted in 141 hospitals in Japan [65]. A total of 1070 patients were included in the intention-to-treat population. The target dose of febuxostat was 40 mg/day, and the allopurinol dose was 100 mg/day. In this study, the development of microalbuminuria, progression to overt proteinuria or worsening of overt proteinuria to ≥ 300 mg/g creatinine was significantly lower in the febuxostat-treated group.

#### Interpreting the Results of Clinical Trials

In the review by Sato et al., clinical trials on ULT in CKD were classified as “interpretable” or “non-interpretable” based on whether the control group also showed evidence (or not) of clinically relevant progression of CKD, considering a threshold value of ≥ 4 ml/min/1.73 m^2^ over the course of the study [42]. Of the 22 studies identified, 14 were considered as “interpretable” as they documented progression of CKD in the control group, while the remaining were “non-interpretable” since the control groups either showed no progression of CKD or the progression was less than the defined threshold. All of the interpretable studies showed a benefit of ULT on slowing progression of CKD compared with the control group, compared with none in the non-interpretable group. On this basis, the authors concluded that this provides some evidence for the benefits of ULT in slowing the decline in renal function in patients with CKD and hyperuricemia, which is likely to be especially relevant in those with stage 3 CKD or higher.

#### Febuxostat vs. Allopurinol

More recently, several authors have compared ULT with either febuxostat or allopurinol in patients with CKD. Interestingly, most trials reported that febuxostat is superior to allopurinol in delaying progression of CKD. A retrospective review of medical records of adult patients was made by Lee et al. in 141 patients with stage 3 CKD and hyperuricemia who openly received either allopurinol or febuxostat and were followed from 2005 to 2018 [66]. Patients on febuxostat, compared with allopurinol and controls, had significantly lower mean serum uric acid levels (5.7 vs. 7.1 vs. 8.0 mg/dl, *p* < 0.001) and maintained significantly higher mean eGFR values for 4 years. Patients in the febuxostat group also had significantly longer renal survival time free from progression of renal disease than allopurinol and controls (87.7 vs. 77.6 vs. 48.7 months, respectively, *p* < 0.001).

Zhang et al. investigated the progression of renal impairment in a prospective cohort of 152 CKD stage 2–3 patients with hyperuricemia [67]. In this cohort, patients received either allopurinol (maximum dose of 200 mg/day) or febuxostat (maximum dose of 40 mg/day). Interestingly, patients selected the suitable ULT regimen according to their personal willingness of themselves and their clinical status. After 6 months, eGFR was higher in those treated with febuxostat; patients treated with febuxostat also showed an increase in eGFR compared with allopurinol (+ 4.62 vs. – 0.42 ml/min/1.73 m^2^). The proportion of patients showing a ≥ 10% decline in eGFR from baseline at 6 months was also lower with febuxostat 17.9% vs. 34.1%). The retrospective study by Yang involved 83 patients on allopurinol and 233 patients on febuxostat and similarly reported that the long-term eGFR slope was positive in the febuxostat group and negative in the allopurinol group [68]. Thus, these encouraging results suggest that febuxostat might lead to improved renal function, at least in some patients. However, the quality of the studies comparing allopurinol and febuxostat remains relatively poor with a lack of randomization, a small number of subjects, and thus a relatively low statistical power.

Lastly, febuxostat has been compared with allopurinol in pre-dialysis patients with stage 5 CKD in a large insurance database analysis from Taiwan in over 6000 patients (69.5% allopurinol, 42% febuxostat) [69•]. Median follow-up time was 0.72 years, during which significantly fewer patients treated with febuxostat initiated long-term dialysis (43.56% vs. 65.17%, *p* < 0.0001). Multivariate Cox analysis showed febuxostat was associated with significant risk reduction for long-term dialysis compared with allopurinol (adjusted HR, 0.65; 95% CI, 0.60–0.70). Considering a composite outcome of long-term dialysis or death, 52.72% of those on febuxostat reached that endpoint compared with 81.92% of those on allopurinol (*p* < .0001). Moreover, no significant increase in cardiovascular mortality was observed for patients on febuxostat (adjusted HR, 1.06; 95% CI, 0.76–1.50), and febuxostat was associated with a lower risk of myopathy (adjusted HR, 0.67; 95% CI, 0.53–0.84). As discussed above for earlier CKD stages, these interesting observations would need to be confirmed in a randomized controlled study.

### ULT for Cardiovascular Protection in CKD

Many studies have found serum uric acid to be an independent cardiovascular risk factor associated with a higher risk of events, although there is some debate about whether this relation is due to its association with established cardiovascular risk factors [70]. In recent European hypertension guidelines, uric acid has been added in the list of factors that can increase the risk of cardiovascular events in hypertension [71]. A large meta-analysis of 24 studies on 25,453 patients with CKD found that elevated serum uric acid predicted the risk of mortality, an increase of 1 mg/dl in serum uric acid being associated with an 8% increased risk of mortality [26]. The same authors also carried out a recent meta-analysis on cardiovascular mortality on 11 studies including 27,081 patients with CKD [72]. Based on 7 of these studies, i.e., 10,050 subjects, patients with the highest levels of serum uric acid had an increased risk of cardiovascular mortality (HR 1.47, 95% CI 1.11–1.96) compared with those with the lowest levels. In addition, in another subset of 10 studies, each 1 mg/dl increase in serum uric was associated with an increased risk of cardiovascular mortality by 12%. Notwithstanding, the authors concluded that specifically designed, randomized controlled trials are needed to provide a definitive answer to the question of whether ULT can be of benefit to lower cardiovascular risk in patients with CKD.

### Distilling the Evidence for Daily Practice

There is now increasing evidence to suggest that elevated levels of serum uric acid are associated with both CKD and cardiovascular risk. However, there is large heterogeneity in study designs, endpoints, and baseline characteristics making it very difficult to compare the results of multiple trials and to draw firm conclusions. For example, a recent study in patients with type 1 diabetes and early-to-moderate diabetes kidney disease found that there were no clinically meaningful benefits of ULT on renal outcomes [73•]. Another recent trial randomized patients with stage 3 or 4 kidney disease, with no history of gout, to allopurinol or placebo and reported that ULT did not slow the decline in eGFR when compared with the placebo group at week 104 [74••]. Notwithstanding, there is still compelling evidence that links CKD to uric acid, which has an injurious impact on renal function. In this regard, a “two-hit” mechanism has been proposed involving activation of the renin-angiotensin system and inhibition of nitric oxide synthesis, leading to a small increase in blood pressure, and involvement of the inflammasome and subsequent secretion of proinflammatory cytokines [73•]. In such a mechanism, ULT in patients with CKD and hyperuricemia with or without deposition has the potential to slow or delay the progression of CKD and may be considered unless there are clear contraindications to ULT. Most guidelines provide clear recommendations and targets for ULT in patients with hyperuricemia with deposition and/or gout (reviewed in [75]). However, formal guidance is much less clear in patients with CKD and hyperuricemia without deposition.

In a recent expert review, Sato et al. note that as ULT is not approved for treatment of CKD, any treatment decisions must be made following shared discussion with the patient [42]. Dietary modification should also be used to lower serum urate. However, as diet is likely to be effective by itself, especially in CKD, these authors recommend that xanthine oxidase inhibitors should be considered as the primary class of ULT for patients with CKD, and thus mainly allopurinol and febuxostat. Of note, according to the respective Summaries of Product Characteristics, in patients with CKD, dose reduction may be needed with allopurinol; for febuxostat, no dose reduction is needed for mild to moderate renal impairment, and it is noted that febuxostat has not been well studied in patients with severe renal impairment (creatinine clearance < 30 ml/min).

Regarding target levels, it has been proposed that ULT should be initiated in patients with serum urate ≥ 7 mg/dl (416 μmol/l), with a target of < 6 mg/dl (357 μmol/l). What should be done in those with intermediate values remains unclear [76]. There is some discrepancy on whether or not ULT should be administered in the absence of progression of CKD, with some in favor of ULT only when clinically relevant progression is observed [42] and others advocating that all hyperuricemic patients with CKD can benefit from ULT [57]. However, once progression occurs, it is likely that damage to the kidney has already taken place, and thus early intervention might be considered with a preventive aim. In this regard, recent trials with febuxostat have shown that it may even be associated with improvement in renal function. With this in mind, it would not appear acceptable to deprive patients of a therapy that may have the potential to improve their renal function unless there are contraindications for doing so. Nonetheless, it is recognized that further studies are needed to better understand in which patients, at which stage of CKD, and the preferred ULT in this population. In patients with repeated gout episodes, ULT should be prescribed in any case whatever the renal function.

Considerations for the various ULT used with a focus on CKD stages 3–5 are shown in Table 1.

**Table 1:** Considerations for urate-lowering agents in CKD stages 3–5

Urate-lowering agent	Recommendations for CKD 3–5
Allopurinol	• eGFR ≥ 30 ml/min/1.73 m^2^: start with ≤ 100 mg/d • eGFR < 30 ml/min/1.73m^2^: start with 50 mg/d
Febuxostat	• Insufficient data for eGFR < 30 ml/min/1.73 m^2^
Colchicine	• Not recommended in patients already receiving colchicine for prophylaxis • eGFR ≥ 30 ml/min/1.73 m^2^: dosage adjustment not required • eGFR < 30 ml/min/1.73 m^2^: consider dosage reduction; treatment course should not be repeated more frequently than every 14 days
NSAID	• eGFR 30–59 ml/min/1.73 m^2^: use with caution or avoid depending on the kidney disease • eGFR < 30 ml/min/1.73 m^2^: relatively contraindicated
Glucocorticoid	• Dosage adjustment for CKD not required
ACTH	• Dosage adjustment for CKD not required
Interleukin-1 inhibitors	• eGFR < 30 ml/min: for anakinra, mean plasma clearance of anakinra declined by 70–75%; consider dose reduction, as 100 mg every other day; for canakinumab, no dose reduction is needed, though clinical experience is limited

*eGFR* estimated glomerular filtration rate as calculated by CKD-EPI formula

## Conclusions

There is growing evidence that hyperuricemia with and without deposition is associated with high cardiovascular risk and a decline of renal function. Indeed, there is now an abundance of experimental and clinical data suggesting that hyperuricemia may promote and accelerate kidney damage through several well-understood mechanisms. Many clinical studies have provided support to the hypothesis that ULT may be beneficial in preventing and delaying a decline of renal function in patients with CKD. While the observed discrepancies in the outcomes of clinical trials on ULT in patients with CKD may indicate that not all patients will receive renal benefits of ULT, the discrepancies may also be due to a number of other factors, such as low sample size, short duration of follow-up, and lack of consistent definitions of CKD across studies as well as heterogeneity in study designs. Although current guidelines for management of CKD do not recommend treatment of hyperuricemia in the absence of a diagnosis of gout [75], there is increasing consensus that there is a direct causal relationship between high levels of serum urate and the development and progression of CKD [42, 57]. ULT with a xanthine oxidase inhibitor should be considered as a therapeutic option in patients at high renal risk and/or declining renal function with hyperuricemia with and without deposition, even if additional studies are needed to identify threshold values for treatment and treatment targets. The possibility to delay deterioration of renal function in patients with CKD represents an opportunity that should be discussed with patients. In this regard, comparative studies suggest that febuxostat is more effective than allopurinol in halting progression of CKD, but larger randomized double-blind controlled studies are needed to confirm this advantage of febuxostat.
